# Hall’s Medical Adviser

**Published:** 1875-02

**Authors:** 

**Affiliations:** 234 Broadway, New York


### HALL'S MEDICAL ADVISER,

**EDITED BY DR. W. W. HALL.**

Published Monthly at 234 Broadway, New York,

(Opposite the New Post Office).

Thirty-two pages, 8vo. $1 a year; Single number, ten cents; Six copies for five dollars; Fifteen copies for ten dollars.

All articles will be written by the Editor. The object is to give, at a low rate, such information as to habits, modes of life, dress, exercise, air, etc., as been found in the observation and experience of eminent medical men best calculated to

PRESERVE HEALTH

and also to cure many maladies which really do not require medicine, amounting, perhaps, to three-fourths of all ordinary diseases. A main object of the Editor is to afford a cheap vehicle of information about health to

THEOLOGICAL STUDENTS, and, next to them, to young girls who may, in the future, be dependent on their own efforts for a living, and also for

HOUSEHOLDS, for the use of grown-up children, and for the guidance of mothers in raising their little ones healthily; also, for all the young at school, who by a little information may preserve their health, but for the want of it may lose that health early and be invalids for life, comparatively useless to themselves, if not indeed a burden to others.

To those who wish to present the ADVISER to absent children, to friends, to clergymen, and to public institutions and libraries, twenty copies will be sent to as many addresses for one year for ten dollars, sent at one time.

TO NEWSMEN, who wish to sell again, ten copies or more will be sent for six cents per copy, post paid.

The ADVISER will be sent with any three, four, or five dollar newspaper or magazine or other periodical at the price of the latter; thus two publications will be sent for the price of one.

The ADVISER is not intended to take the place of "Hall's Journal of Health," for the Editor is personally and pecuniarily interested in its large success, but it is felt that very, very many would give a dollar a year for a periodical which treated health matters only, who would not feel able or willing to give two dollars for one which gave also a large amount of valuable miscellaneous reading.

The Editor's office and rooms for medical consultation are at 234 Broadway, New York, opposite the New Post Office, and near the Astor House from ten to three daily; where also may be had all his publications, to wit: "Health by Good Living," "Sleep; or, Hygiene of the Night," "Health and Disease," or *constipation*, its nature, cause, effects, and cure without medicine; "Bronchitis and Kindred Diseases;" "Consumption," being narrations of eminent physicians of its arrest and permanent cure by out-door activities. Bound vols. of "Hall's Journal of Health" for twenty-one years. All the above sent for $1.50 per vol., or either one for five subscriptions. "Health at Home;" or, Hall's Family Doctor, 850 pp., $5, will be sent for ten subscriptions; a most useful book to all families in limited circumstances or who live remote from physicians, and especially to the newly married, as it not only gives minute information to young mothers about rearing children from the hour of birth to maturity, but it shows how, with reasonable uniformity, children may be born with but little suffering and with good health and high mental and moral traits of character. Examples given as proof.

